# Bcl-2 together with PI3K p110*α* regulates cell morphology and cell migration

**DOI:** 10.1038/cddis.2015.345

**Published:** 2015-12-03

**Authors:** G Wan, A Mahajan, D Lidke, A Rajput

**Affiliations:** 1 Division of Surgical Oncology, Department of Surgery, University of New Mexico, Albuquerque, NM, USA; 2 Department of Pathology, University of New Mexico, Albuquerque, NM, USA

The signaling network of phosphatidylinositol 3-kinase (PI3K) and Akt controls cell cycle, survival, metabolism and genomic instability. It is also involved in cell motility and cancer metastasis.^1^ Genetic mutations of PI3K frequently happen in various cancers, induce PI3K dysfunction and result in increased cell migration and cancer metastasis.^2^ Involvement of the PI3K-Akt pathway in the anti-apoptotic function of B-cell lymphoma 2 (Bcl-2) has been well defined. Previous work has also shown that activated PI3K results in phosphorylation of Akt, whereas the activated Akt in turn upregulates Bcl-2 by enhancing promoter activity of Bcl-2.^3^ These findings suggest that PI3K is involved in the regulation of Bcl-2 expression. The mutation mediated dysfunction of PI3K may alter the regulation of Bcl-2. Our recent work, published in *Cell Death and Discovery*,^4^ shows that Bcl-2 expression is downregulated at least threefold by the most frequent mutation H1047R in the p110*α* subunit of class IA PI3K. We show this by comparing endogenous levels of Bcl-2 in human colorectal cancer (CRC) HCT116 WT and MUT cells that were engineered from parental HCT116 cells to contain either the wild type (WT) or H1047R mutant (MUT)-p110*α*, respectively. This finding was further confirmed by the examination of PI3K p110*α* inhibition using PI3K inhibitor A66, which has greater specificity in inhibiting p110*α* as compared with other p110*α* inhibitors and thus maintains the function of other PI3Ks in growth factor signaling. Inhibition of H1047R-p110*α* results in an A66-dose-dependent increase in Bcl-2 expression. In contrast, inhibition of WT-p110*α* shows an A66-dose-dependent decrease in Bcl-2 expression.^4^ These data suggest that cellular Bcl-2 levels are differentially regulated by the presence of either WT or MUT p110*α*.

In addition to its well-characterized role in the suppression of programmed cell death, Bcl-2 has been associated with cell proliferation, differentiation, mutagenesis, cytoskeletal reorganization, cell migration and cancer metastasis. Data examining the functional role of Bcl-2 in cell adhesion, migration and branching morphogenesis shows that lack of Bcl-2 in ureteric bud cells results in increased cell migration, increased cell invasion and decreased adhesion to vitronectin as compared with WT-ureteric bud cells, and suggests that Bcl-2 is required for the proper regulation of cell adhesive and migratory mechanisms, perhaps through modulation of the cellular microenvironment.^5^ Another group examined the effects of Bcl-2 overexpression on cell morphology of undifferentiated PC12 cells and demonstrated that overexpression of Bcl-2 leads to disruption of the actin cytoskeleton and alteration of cell morphology.^6^ Moreover, Ke *et al.* recently reported that overexpression of Bcl-2 inhibits cell adhesion, spreading and motility by enhancing actin polymerization.^7^ They suggested that when overexpressed in both cancer and non-cancer cells, Bcl-2 can form a complex with actin and gelsolin that functions to decrease gelsolin-severing activity that leads to increased actin polymerization.

Actin polymerization can generate forces that underlie alterations in cellular morphology, protrusion, migration and chemotaxis that occur during morphogenesis.^8, 9^ Cancer cells control their migratory and invasive capability through morphogenic alteration. These processes involve a marked reorganization of the actin cytoskeleton and the concomitant formation of membrane protrusions required for cell motility in a complex three-dimensional environment, including lamellipodia, filopodia, podosomes and invadopodia.^10, 11^ Our data showed that the H1047R mutation in p110*α* of PI3K decreases actin polymerization, increases filopodia formation, and results in cell morphology changes in HCT116 cells (Figure 1a). Interestingly, H1047R mutation in p110*α* of PI3K downregulates Bcl-2, whereas the morphology of HCT116 MUT cells was altered when Bcl-2 was overexpressed (Figure 1b). Based on the aforementioned reports, the H1047R mutation mediated downregulation of Bcl-2 may provide an explanation for why the H1047R mutation in p110*α* can induce reorganization of actin cytoskeleton, and thus results in morphological changes and increased migratory capability in HCT116 MUT cells. The distinct effects of PI3K on regulation of Bcl-2 and actin cytoskeleton, however, resulted from either the presence of WT or MUT p110*α*. This suggests that WT and H1047R MUT p110*α* of PI3K may regulate actin cytoskeleton and cell migration by cooperation with Bcl-2 through distinct molecular mechanisms.

Overexpression of Bcl-2 occurs in many types of human cancers, and prevents cell death induced by nearly all anticancer drugs and radiation. The functional roles of Bcl-2 in tumor development and progression or metastasis, however, are quite unclear and often contradictory. Several reports have indicated that Bcl-2 increases tumor progression in some types of cancer. On the other hand, data from previous *in vivo* studies have shown that loss of Bcl-2 expression correlates with tumor recurrence in CRC^12^ and high levels of Bcl-2 are predictive of relapse-free survival in stage II CRC.^13^ Clinical observations reporting that Bcl-2 expression in breast cancer can be associated with a favorable prognosis suggests a possible beneficial role for Bcl-2 in suppressing tumor progression and metastasis.^14^ Using an *in vitro* wound healing assay, we showed that H1047R-p110*α* increases migratory capacity of HCT116 cells. The cell migration, however, was slowed down in HCT116 MUT cells when Bcl-2 was stably overexpressed.^4^ To note, a recent study shows that knockdown of Bcl-2 proteins directly inhibits the migration and invasion of the CRC cells HT29 and SW480, independent of their cell death induction or effects on proliferation.^15^ These contradictory effects of Bcl-2 overexpression on cell migratory capability seen in different CRC cell lines may indicate the importance of cellular environment, for example the presence of different types of PI3K p110*α*. It is known that SW480 cell line expresses WT PI3K and that HT29 cells bear the P449T^b^ mutation in p110*α*. All of these observations further suggest the multiple and complex functions of Bcl-2 and PI3K.

In conclusion, although Bcl-2 functions as an oncogene to prevent programmed cell death and promotes tumorigenesis, our study has shown that high levels of Bcl-2 may also prevent tumor metastasis. This function is probably due to the ability of Bcl-2 to regulate actin polymerization in a way that inhibits cell migration. Moreover, Bcl-2 may be differentially regulated by PI3K depending on the presence of WT or MUT p110*α* that may activate distinct signaling pathways and differentially control cell migratory capability and cancer metastasis (Figure 1c). Our work links the MUT and WT types of PI3K p110*α* and Bcl-2 in controlling cytoskeleton rearrangement, migratory capability of CRC cells and CRC metastasis. This may provide a novel concept for performing studies on molecular mechanisms involved in cancer metastasis and a possible biomarker development for predicting cancer metastasis.

## Figures and Tables

**Figure 1:**
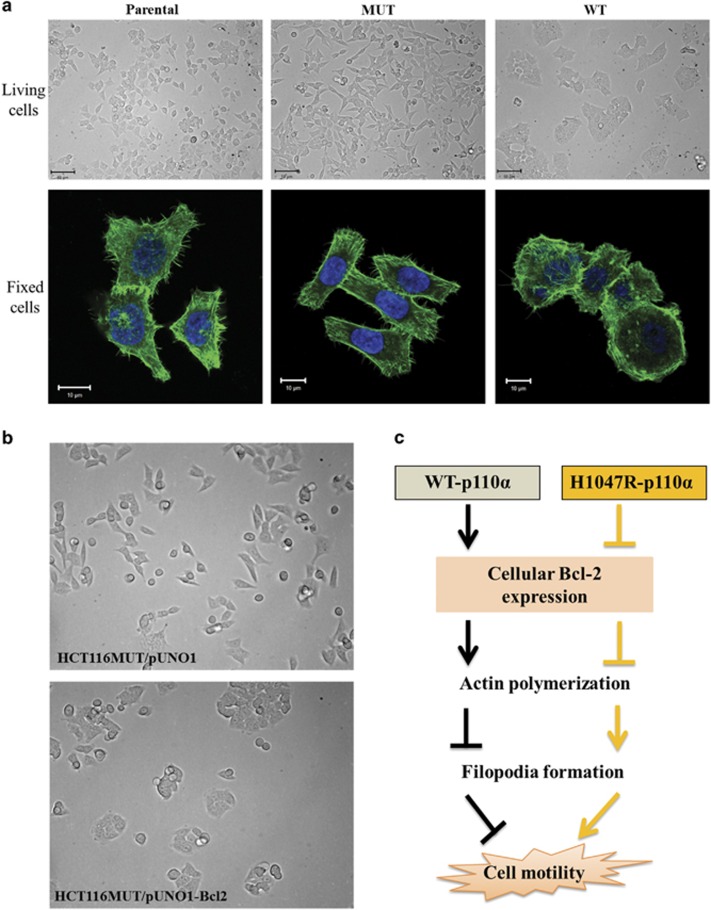
Cell morphology and cell migration of HCT116 cells are altered by the H1047R mutation in the p110*α* kinase domain of PI3K and Bcl-2. (**a**) Cell morphology of HCT116 cells. Top panel: cell morphologies of live parental, WT and MUT HCT116 cells captured at a 20 × magnification. Bottom panel: confocal images of parental, WT and MUT HCT116 cells captured at a 63 × magnification. Cells were fixed and stained for F-actin (green). Nuclei were stained with DAPI (blue). (**b**) Overexpression of Bcl-2 changed cell morphology of HCT116 MUT cells. HCT116 MUT cells were stably transfected with the pUNO1 or pUNO1-Bcl-2 plasmid and imaged at 20 × magnification, the morphology of cells changed as they became rounded and aggregated together when Bcl-2 was overexpressed. (**c**) Model for the cooperative role of Bcl-2 with WT or H1047R -p110*α* to control cell motility in HCT116 cells. The symbol ⊥ means decrease and ↓ means increase. The H1047R mutation in p110*α* causes the downregulation of Bcl-2, which decreases actin polymerization, induces reorganization of actin cytoskeleton
